# A Rare Case of Intermittent Claudication Associated with Impaired Arterial Vasodilation

**DOI:** 10.1155/2017/4868123

**Published:** 2017-12-24

**Authors:** J. J. Posthuma, K. D. Reesink, M. Schütten, C. Ghossein, M. E. Spaanderman, H. ten Cate, G. Schep

**Affiliations:** ^1^Laboratory for Clinical Thrombosis and Haemostasis, Department of Internal Medicine, Cardiovascular Research Institute Maastricht, Maastricht University Medical Centre, Maastricht, Netherlands; ^2^Department of Biomedical Engineering, Maastricht University Medical Centre, Maastricht, Netherlands; ^3^Department of Internal Medicine, Cardiovascular Research Institute Maastricht, Maastricht University Medical Centre, Maastricht, Netherlands; ^4^Department of Obstetrics and Gynecology, Maastricht University Medical Centre, Maastricht, Netherlands; ^5^Department of Sports Medicine, Máxima Medical Centre, Veldhoven, Netherlands

## Abstract

Exercise-related intermittent claudication is marked by reduced blood flow to extremities caused by either stenosis or impaired vascular function. Although intermittent claudication is common in the elderly, it rarely occurs in the young and middle-aged individuals. Here, we report a case of exercise-related claudication in a 41-year-old woman, in the absence of overt vascular pathology. Using a series of imaging and functional tests, we established that her complaints were due to impaired arterial vasodilation, possibly due to a defect in nitrous oxide-mediated dilation. The symptoms were reversible upon administration of a calcium antagonist, showing reversibility of the vascular impairment. Identification of reversible vascular “stiffness” merits consideration in young and otherwise healthy subjects with claudication of unknown origin.

## 1. Introduction

Intermittent claudication is characterized by cramp-like pain in the leg upon exertion, due to insufficient blood flow. Typically, intermittent claudication affects the elderly, most often due to intraluminal stenosis in the peripheral arteries, resulting from atherosclerosis. Intermittent claudication is the commonest presentation of peripheral arterial disease in the elderly [1] but is rare in the young. In younger subjects (<45 years), intermittent claudication may be associated with specific forms of exercise, for example, cycling [2]. Such exercise-related claudication is often due to anatomical malformations such as popliteal artery entrapment or endofibrosis. Here, we present a case of exercise-related claudication in a young woman, apparently associated with reduced arterial vasodilation, in the absence of overt vascular pathology.

## 2. Case Description

A 41-year-old white European woman presented to our clinic with a 7-year history of unexplained progressive unilateral left thigh pain during cycling, starting at moderate intensity and increasing upon maximal exertion. She had been a spinning instructor for 13 years, with an average of 5 hours per week, before onset of symptoms. In addition, she worked as a firefighter, where she experienced identical complaints during rapid actions, like climbing the stairs. The patient reported a sensation of unilateral muscle fatigue and pain in the left thigh, starting at the vastus medialis and spreading all over the quadriceps, adductors, and biceps femoris, without symptoms in the gluteal region or calf. Complaints vanished within 3 minutes after cessation of exercise and were highly reproducible. There was no history of trauma, while her medical history included right-sided congenital hip dysplasia, primary Raynaud's phenomenon, and attention deficit hyperactivity disorder (ADHD), for which she used clomipramine for 10 years. She was on no other drug therapy at time of presentation and had no history of smoking, diabetes, hypertension, hypercholesterolemia, peripheral artery disease, or coronary heart disease. Family history was positive for familial hyperhomocysteinemia, but this was never diagnosed in her.

### 2.1. Clinical Examination

The woman presented with a height of 172 cm and body weight of 65 kg (body mass index of 22 kg/m^2^). Her resting blood pressure was 120/75 mmHg with a heart rate of 61 beats per minute (bpm). Musculoskeletal investigation showed normal back mobility and a full range of motion of hips and knees with normal muscular strength of both legs. In addition, neurological examination was normal in our patient.

### 2.2. Laboratory Evaluation

Admission laboratory findings included complete blood count, which was unremarkable (Table 1). In addition, haemostatic tests, including activated partial thromboplastin time (aPTT), prothrombin time (PT), INR, fibrinogen, D-dimer, DNA testing for Factor V Leiden and prothrombin G20210A carriership, protein C activity, activated protein C resistance, and free protein S level, were all normal, while no lupus anticoagulants or anti-cardiolipin antibodies were detectable.

### 2.3. Cardiac Examination

Transthoracic echocardiography showed normal anatomy with normal left ventricular ejection fraction (LVEF) and systolic and diastolic volumes but suggested a slight increase in total peripheral vascular resistance (Table 2).

### 2.4. Exercise Test

Since symptoms appeared during exercise, a maximal treadmill exercise test was obtained with increasing resistance, starting at 100 Watt and elevating 15 Watt every minute, until exhaustion. Complaints started at 130 Watt and the test was ceased at 260 Watt due to unilateral thigh pain and muscular fatigue. The exercise electrocardiogram (ECG) showed no abnormalities and systolic pressures were obtained before and after exercise from the left brachial and bilateral calf for ankle-brachial index (ABI) calculation. The ABI before exercise was 1.1 on both sides, whereas directly after exercise ABI was reduced for both legs (right: 0.75; left: 0.59), where no alterations upon exercise were expected in a healthy young woman [3] (Table 3).

### 2.5. Vascular Examination

Prompted by bilateral reduced ABI after exercise and the suggested increased vascular resistance, further vascular examinations were performed. Normal lower limb pulses without audible murmurs and a normal capillary refill (<2 sec) were found in both legs. To investigate whether this patient suffered from endofibrosis, resting arterial duplex ultrasonography was done, which revealed no stenosis and normal diameters of the external iliac artery and common iliac artery, where femoral arteries appeared to be relatively wide, as might be expected in well-trained individuals [4] (Table 4). In addition, femoral intima-media thickness was within normal range (0.78 mm, reference cut off: <0.9 mm), accompanied by normal peak systolic velocity during hip extension (1.41 m/sec) and flexion (1.54 m/sec) [5]. At the venous level, no signs of (previous) deep vein thrombosis were observed.

Additionally, obstructive vascular pathologies were investigated by multiphase computer tomography angiography (CTA), showing no signs of intraluminal pathologies or aberrant morphology, thereby corroborating the duplex examinations and excluding endofibrosis, popliteal entrapment syndrome, arterial-venous shunts, and venous malformations.

Next, we evaluated arterial function by ultrasonic examination of carotid and brachial artery and tonometric measurement of carotid-femoral pulse wave velocity (cfPWV). Carotid intima-media thickness (0.68 mm, reference cut off: <0.9 mm [6]) and cfPWV (6.74 m/s, reference cut off: <10 m/s [7]) were unremarkable. However, carotid distensibility coefficient was lower than expected (16 × 10^−3^/kPa, reference value: 20–30 × 10^−3^/kPa) [8].

Brachial flow-mediated dilation (FMD, was 1%, reference value: 3.0–8.4% [9]) and nitroglycerine-mediated dilation, 8%, (reference value: 9.6–18.0% [10]) were markedly reduced. Microcirculatory studies showed normal capillary density before (74/mm^2^, reference value: 40.4–85.6/mm^2^) and after (109 mm^2^, reference value: 69.5–117.1/mm^2^) venous congestion [11]. Furthermore, heat-induced cutaneous microvascular dilatation was within normal limits (+1356%, reference value: 324–1762%).

### 2.6. Therapeutic Strategy

Based on the symptoms and test results, we postulated impaired arterial vasodilation and therapy with isosorbide mononitrate 30 mg twice daily was started, without any effects on the symptoms. This appeared to exclude a lack of nitrous oxide as major underlying defect. In another approach, we prescribed a vasodilating agent, the long-acting calcium antagonist diltiazem 200 mg once daily, through which claudication symptoms were well controlled and blood pressure remained within normal range (24 h measurement: average 110/80 mmHg) without any symptoms of orthostasis. During 2-year follow-up, she experienced no side effects and was able to continue her work as a fire fighter and spinning instructor.

## 3. Discussion

We described the case of a 41-year-old woman who presented with exercise-related intermittent claudication. Although the patient experienced unilateral symptoms, a bilateral diminished blood flow was found after exercise, as shown by reduced ankle-brachial index, with tendency towards worse flow at the symptomatic side. This pointed towards a vascular origin of the symptoms. First of all, clomipramine-induced vasospasm was considered as a potential cause of symptoms. However, this was deemed unlikely, since the patient had been on clomipramine therapy for 3 years before the onset of symptoms. In addition to this, stopping clomipramine for several months had no effects on symptoms. Therefore, the differential diagnosis of vascular-related claudication during exertion included endofibrosis, atherosclerosis, May-Turner syndrome, Williams syndrome, endothelial dysfunction, eNOS deficiency, scleroderma, Leriche's syndrome, exercise-induced vasospasm, popliteal entrapment syndrome, and Takayasu's disease. Most of these diagnoses could be excluded, due to absence of intraluminal stenosis or aberrant morphology on multiphase CTA and unremarkable laboratory results. In addition, our patient lacked symptoms of skin lesions that were characteristic for scleroderma, thereby unlikely the cause of symptoms in our patient. Since the symptoms were suggestive for endofibrosis, a full work-up according to the latest consensus study was performed [12], including duplex ultrasound with extended leg and in provoked position and multiphase CTA imaging, neither of which showed signs of endofibrosis, thereby basically ruling out the probability that endofibrosis was the cause of symptoms in our patient. This was further supported by increased total peripheral vascular resistance and reduced dilatory response of brachial artery to endothelial-dependent and -independent stimuli, which is very unlikely in endofibrosis. In addition, in the absence of other intraluminal pathologies and aberrant morphology on multiphase CTA, the cause of symptoms in this patient appears to be (subclinically) impaired vascular function rather than intraluminal obstruction or anatomical malformation.

Exercise-induced vasospasm was considered as diagnosis but appears unlikely in the presence of normal arterial diameter and normal peak flow velocities [13]. Reduced vascular function is supported by impaired ABI after exercise, increased total peripheral resistance, and increased systemic arterial stiffness as shown by relatively low carotid distensibility coefficient. Additionally, we consider the decreases in brachial artery flow-mediated dilation (FMD) and nitroglycerin-mediated dilation (NMD) to be important. FMD-induced changes in arterial diameter are caused by shear-stress-induced endothelial release of nitric oxide [14]. Nitric oxide is the major substance, mediating vasodilatation by inducing smooth muscle cell relaxation in the arterial media. Therefore, FMD provides insight in the peripheral artery endothelial function (NO release), as well as the responsiveness to NO smooth muscle in the arterial wall. During NMD assessment, NO is supplemented independent of the endothelium. Therefore, the observed reductions in FMD and NMD of the brachial artery suggest functional impairment of smooth muscle in the arterial wall rather than endothelium-related disease such as endothelial dysfunction and eNOS deficiency. Together with the lack of relief of symptoms with isosorbide mononitrate in this patient, the above seems to indicate that the symptoms may be caused by reduced sensitivity of smooth muscle cells for NO or by NO diffusion impairment. This was supported by relief of symptoms upon addition of a calcium antagonist, since calcium can regulate the vascular contractility in an NO-independent way.

## 4. Conclusions

This case report illustrates a rare case of symptomatic claudication, most likely due to reduced arterial vasodilation, most likely due to a reduced sensitivity for nitric oxide. While further study as to its origin is required, we recommend additional functional testing of flow-mediated vasodilation and vascular distensibility coefficient in subjects with inexplicable arterial symptoms in absence of evident anatomical malformations, endofibrosis, or other intraluminal pathologies.

## Figures and Tables

**Table 1:** Laboratory evaluation.

Parameter	Value	Reference range
Hemoglobin (g/dL)	14.18	12–18
Hematocrit (L/L)	0.41	0.36–0.47
Mean corpuscular volume (fL)	96	82–98
Thrombocytes (×10^9^/L)	244	150–400
Leukocytes (×10^9^/L)	5.9	4.5–11
MDRD-eGFR (mL/min/1.73 m)	>60	>60
Creatinine (*μ*mol/L)	80	45–80
C-reactive protein (mg/L)	<1	<10
Erythrocyte sedimentation rate (mm)	2	<30
Total cholesterol (mmol/L)	4.2	1.5–6.5
LDL-cholesterol (mmol/L)	2.3	2.0–4.5
HDL-cholesterol (mmol/L)	1.5	0.9–1.7
Triglyceride (mmol/L)	0.95	0.6–2.2
Cholesterol/HDL ratio	2.8	<8
Total calcium (mmol/L)	2.27	2.10–2.55
Homocysteine (*µ*mol/L)	9.5	<12.2

**Table 2:** Cardiac examination.

Parameter	Results	Reference value
*Transthoracic sonography*		
Left ventricular ejection fraction (%)	70	>55
End-diastolic volume (ml)	103	56–104
End-systolic volume (ml)	31	19–49
Total peripheral vascular resistance (dyne·s/cm^5^)	1621	1200–1600

**Table 3:** Vascular diameter and intima-media thickness.

Parameter	Results		Reference value
	Left	Right	
*Duplex ultrasonography*			
Diameter of distal aorta (mm)	14.5		
Diameter of common iliac artery (mm)	9.8	9.9	7.9–11.7
Diameter of external iliac artery (mm)	7.9	7.8	6.7–9.2
Diameter of femoral artery (mm)	10.5	9.2	7.6–8.9
Carotid intima-media thickness (mm)	0.69	0.61	<0.9
Femoral intima-media thickness (mm)	0.78	0.80	<0.9

**Table 4:** Vascular function.

Parameter	Results		Reference value
	Left	Right	
Ankle-brachial index at rest	1.1	1.1	>0.8
Ankle-brachial index after exercise	0.59	0.75	>0.8
Capillary refill at rest (sec)	<2	<2	<2

*Vascular function*			
Carotid-femoral pulse wave velocity (m/s)	6.74		<10
Peak systolic velocity external iliac artery during hip extension (m/s)	1.41	1.37	0.89–1.41
Peak systolic velocity external iliac artery during hip flexion (m/s)	1.54	1.48	n/a
Carotid distensibility (×10^−3^/kPa^−1^)	16.1		20–30
Brachial flow mediated dilation (%)	1		2.4–8.4
Brachial nitroglycerine mediated dilation (%)	8		20–30

*Cutaneous microcirculation*			
Capillary density before venous congestion (/mm^2^)	74		50.4–85.6
Capillary density after venous congestion (/mm^2^)	109		69.5–117.1
Cutaneous blood flow response to warmth (%)	1356		324–1762
